# Mobilising context as complex and dynamic in evaluations of complex health interventions

**DOI:** 10.1186/s12913-023-10354-5

**Published:** 2023-12-18

**Authors:** Jamie Murdoch, Sara Paparini, Chrysanthi Papoutsi, Hannah James, Trisha Greenhalgh, Sara E. Shaw

**Affiliations:** 1 School of Life Course and Population Sciences, King’s College London, London, UK; 2 Wolfson Institute of Population Health, Queen Mary University of London, London, UK; 3 Nuffield Department of Primary Care Health Sciences, University of Oxford, Oxford, UK

**Keywords:** Context, Complex interventions, Public health, Health systems, Complexity, Complex systems, Case study research

## Abstract

**Background:**

The relationship between healthcare interventions and context is widely conceived as involving complex and dynamic interactions over time. However, evaluations of complex health interventions frequently fail to mobilise such complexity, reporting context and interventions as reified and demarcated categories. This raises questions about practices shaping knowledge about context, with implications for who and what we make visible in our research. Viewed through the lens of case study research, we draw on data collected for the Triple C study (focused on Case study, Context and Complex interventions), to critique these practices, and call for system-wide changes in how notions of context are operationalised in evaluations of complex health interventions.

**Methods:**

The Triple C study was funded by the Medical Research Council to develop case study guidance and reporting principles taking account of context and complexity. As part of this study, a one-day workshop with 58 participants and nine interviews were conducted with those involved in researching, evaluating, publishing, funding and developing policy and practice from case study research. Discussions focused on how to conceptualise and operationalise context within case study evaluations of complex health interventions. Analysis focused on different constructions and connections of context in relation to complex interventions and the wider social forces structuring participant’s accounts.

**Results:**

We found knowledge-making practices about context shaped by epistemic and political forces, manifesting as: tensions between articulating complexity and clarity of description; ontological (in)coherence between conceptualisations of context and methods used; and reified versions of context being privileged when communicating with funders, journals, policymakers and publics.

**Conclusion:**

We argue that evaluations of complex health interventions urgently requires wide-scale critical reflection on how context is mobilised - by funders, health services researchers, journal editors and policymakers. Connecting with how scholars approach complexity and context across disciplines provides opportunities for creatively expanding the field in which health evaluations are conducted, enabling a critical standpoint to long-established traditions and opening up possibilities for innovating the design of evaluations of complex health interventions.

## Background

*“a claim about context is precisely that—an articulation concerning a set of connections and disconnections thought to be relevant to a specific agent that is socially and historically situated, and to a particular purpose”* [1]

Debates surrounding the meaning of context in evaluations of complex health interventions have a long and contested history. Varying theoretical perspectives driven by distinct disciplinary and epistemic traditions have shaped how context has been conceptualised and subsequently investigated, ranging from constructions of context as a fixed object set within an observable external reality, to context as emergent, relational and boundless [2, 3]. Decades of theorising on the relationship between context and diffusion of innovations [4–6] has directly informed these debates, leading to conceptualisations of interventions and context engaged in complex and dynamic interactions over time, inextricably linked through the social practices of intervention delivery.

However, evidence suggests that operationalising this complexity and dynamism within these health evaluations is rare. Instead, empirical studies have frequently situated context as distinct from the intervention itself, constructed as a bounded epistemic artefact to be understood on its own merits for how it shapes healthcare implementation. The reasons why context has come to be positioned in this way are multi-faceted, but include historical roots in the evolution of objectivity as an “epistemic virtue” in scientific practice, evolving over centuries [7]. Placing a universal, standardised human body centre stage, medical research constructed an epistemology with methods oriented towards finding causal pathways that could be validated, generalised to populations and established as best practice. This same set of conceptual relations is clearly still evident in contemporary empirical health research, which has attempted to answer questions about the effectiveness or cost-effectiveness of complex healthcare interventions or service delivery and how they can be standardised and replicated. Within this perspective, randomised controlled trials are seen as the gold standard of evidence-making practices [8]. Underpinned by assumptions of linear causality, context has almost exclusively been positioned as an external problem to be solved so the intervention can be reproduced to achieve the same effects, and thus constructed as specific confounding variables to be defined, measured and controlled.

This gap between theoretical and empirical knowledge about context prompts us to think carefully about why complex and dynamic understandings of context are rarely mobilised within health evaluations, and the social forces which shape constructions of context throughout the research process. It challenges health researchers to reflect on the decisions they make about what is and what is not relevant context in their research, who gets included and excluded, which phenomena they choose to focus on and how they study them. These questions are not trivial. Decisions about context determine which type of knowledge we make visible to others and which knowledge is hidden, with implications for questions of representation, marginalisation and social justice.

The purpose of this article is to tackle these issues head on through the lens of case study research. Case studies involve in-depth exploration of social phenomena within their ‘natural’, or real-life settings with the potential to provide understanding of dynamic and complex relationships over time [9]. In evaluations of complex health interventions, case studies provide a means of understanding unfolding complex and dynamic context-intervention interactions. In focusing on how context is formulated within case study evaluations of complex health interventions, our aim in this article is to facilitate a much a wider debate about how researchers engage with context using a range of theoretical and methodological approaches, including case studies but also health evaluations adopting other research designs. Importantly, we urge health researchers to carefully consider how they conceptualise and operationalise context when designing and conducting evaluations and call for them to find new ways to connect with how scholars approach complexity and context across disciplines. If the intention is to understand complexity and dynamism in context-intervention interactions, then what system-wide changes are required in how the notion of ‘a context’ is mobilised within evaluations of complex health interventions? We sought to answer this question as part of the Triple C study, funded by the UK Medical Research Council (MRC). Triple C had an overall objective to develop guidance and standards for reporting Case study research into the influence of Context in evaluations of Complex health interventions, and understanding how to mobilise context within case study evaluations was an important element of this work. We did so by opening a dialogue with leading experts - researchers, policymakers and healthcare professionals - at the forefront of debates about context in healthcare research.

This article comes in three parts. Firstly, in the background section, we present a narrative review of how context has been understood and operationalised within published evaluations of complex health interventions. Secondly, we report on the design, methods and findings from the Triple C study. Drawing on our discussions with experts and using illustrative examples, we consider what types of knowledge different versions and uses of context afford and constrain, as well as the forces that shape knowledge ‘about context’ from health evaluations. Finally, in our discussion we call for greater reflection on how health service researchers mobilise context as purposeful knowledge-making practices, and argue that this requires transparency about which knowledge is made visible or invisible and for which purposes. At the heart of our argument is a consideration of how notions of context might be operationalised to generate an understanding of complex and dynamic context-intervention interactions over time. To support this, we draw on ideas about context across disciplines with the potential to creatively extend and expand our thinking about context. These present possible alternatives for ‘doing visibility’ within health evaluations, dispensing with context-as-a-thing as part of the analytical vocabulary, and respecifying context with ontologies which reshape the visibility of the social phenomena we are trying to understand.

### Ways of conceptualising and operationalising context within research of complex health interventions

A diversity of disciplines, including medical sociology, anthropology and sociolinguistics have provided a long history of research across a diverse body of literature which powerfully illustrates the contextualised social practices of health and illness. Such work highlights the dangers of disaggregating context from the actions and interactions in which health and illness is lived and experienced, as well as how health care is delivered and received. Cohn [10] argues that reifying context as a distinct category functions to preclude a sociology of context out of what people actually do and why, as well as naturalising the methods used to carry out such investigations. Yet conceptualising context as a distinct analytical category is a widespread practice in research of complex interventions. This is none more evident than in the emergence of Implementation Science which has produced a proliferation of models and frameworks incorporating context as a central concept, guiding researchers to identify contextual determinants of healthcare delivery [11–13], develop implementation strategies, evaluate intervention implementation within different settings, and to inform the process of translating research findings into practice. Context has been bound within organisations, as ‘inner’ and ‘outer’ context [12], positioned simultaneously as a ‘*thing*’ or factor, and as particular *processes* or characteristics of delivery, as well as broadened to incorporate political, economic and socio-cultural *forces* shaping intervention delivery [13].

Mirroring criticisms of complexity constructed as a set of interacting components [14, 15], formulations of context comprising categorical domains and distinct from the intervention have been challenged as an arbitrary separation offering limited utility, failing to reveal how interventions and ‘their’ context are inextricably linked when enacted through the social practices of intervention delivery [16, 17]. Instead, a different ontology of context is proposed, as situated social actions and interactions, with implications for the type of knowledge produced from individual studies [17–20]. Through this lens, instead of interventions viewed as bounded objects functioning differently under different contextual conditions, the nature of the intervention and context is much harder to pin down, emerging and changing through the ongoing social actions and interactions of doctors, nurses, patients, managers, policymakers who bring that intervention (and context) into being [17].

These differing perspectives of the intervention-context dynamic are reflected in numerous iterations of MRC guidance on developing and evaluating complex health interventions [21–25]. Definitions of context have shifted from “anything external to the intervention that may act as a barrier or facilitator to its implementation, or its effects” [22] to “any feature of the circumstances in which an intervention is conceived, developed, implemented and evaluated” [25]. Accompanying these redefinitions has been an increasingly explicit recognition of complexity and dynamism, positioning interventions as “events within complex systems” [20], shaped and interacting with wider cultural, political, social and economic forces that have a bearing on the social practices of implementation.

However, to date this emergent and dynamic view of context is uncommon in reports of evaluations of complex health interventions. Shoveller, et al’s., [2] critical examination of population health interventions found context frequently being treated as a ‘black box’ or something to be ‘controlled for’, with few studies providing in-depth descriptions of the relationship between context, intervention and outcomes. Similarly, in a discussion of how context has been mobilised within systematic reviews and guidelines, Booth et al., [26] found that despite huge diversity, context was typically bounded within the immediate organisational space and time in which the intervention was embedded, rather than a broader appreciation of how interventions are shaped by social, political or economic forces. In a review of realist evaluation and synthesis, Greenhalgh and Manzano [27] identified two broad narratives which speak directly to these different uses of context. The first narrative framed context as observable triggers (space, place, people, things) operationalised with the assumption that contextual features can be reproduced to optimise intervention implementation; the second framed context as relational and dynamic, implying that it is infinite and uncontrollable.

These twin narratives draw out essentialist and relativist positions that can also be traced across disciplines outside of health research. Using a ‘transdisciplinary’ approach, Dilley [1] threaded the origins of perspectives of context across anthropology to linguistics, literary studies and philosophy. In doing so, Dilley identified how a dilemma of context has emerged, caught within a “hermeneutic circle” of interpretation, between context as external object and context as situated social practice, where social life “derives its meaning from the context or horizon within which it stands; yet the horizon is made up of the very elements to which it gives meaning’’ [28]. The same dilemma was evident in our own review [29] of empirical case studies in health research where we found a significant body of research which described context as a distinct object, fixed in the time and place of observation, and a rarity of studies which reported operationalising context as situated social actions, interactions or practices. We identified four different formulations of context as: 1) characteristics of the implementation setting or human factors impacting the intervention; 2) dynamic organisational, policy or human backdrop, changing over time as the intervention is implemented; 3) a set of circumstances where particular mechanisms are triggered to produce particular outcomes; and 4) emergent and co-shaped through relationships and wider social influences on implementation practices. Importantly, we found a predominance of interview and thematic analytical methods, with very limited critical reflection on the suitability of these methods for empirically facilitating an understanding of interventions and context in complex and dynamic interactions over time.

These findings illustrate how particular versions of context are made visible within evaluations of complex interventions whilst others remain hidden, with implications for the knowledge produced as a result. Using the ‘irreductionist’ program in science and technology studies as their point of departure, Asdal and Moser [30] illustrate the context*ualising* and decontext*ualising* work in which ‘evidence’ is made, and the researchers’ purposive role in that reductive process. Rather than developing pre-defined procedures, frameworks or typologies for investigating context-as-a-thing, Asdal and Moser propose “experimenting” with making context, in what they term “contexting.” We now apply these ideas to our discussions with experts in the Triple C study, framing individual’s talk as a negotiation of Dilley’s dilemma as contexting practices, where speakers connect and disconnect versions of context and interventions, shaping what is made visible from healthcare evaluations.

## Methods

Triple C was commissioned by the UK MRC Better Methods, Better Research panel, partly in recognition of the limited ability of randomised controlled trials to address questions of causality within complex systems and the need to support methods that enable insights into the delivery of healthcare interventions in conditions of complexity [9, 29, 31]. Case study research offers such potential, providing a means of investigating social phenomena which embraces, rather than attempts to control, the influence of context in the life of interventions. However, case study approaches have been under-utilised and poorly reported in healthcare research, including a lack of clarity of how both ‘the case’ and ‘the context’ have been theorised and operationalised within empirical studies [9].

The overall aim of the Triple C study was to develop case study guidance and reporting principles [31], taking account of context and complexity. Study methods involved a) a meta-narrative review on how case study approaches have been used in health research, (of which we have summarised findings in the background section); b) interviews with researchers, journal editors, healthcare professionals and policymakers; and c) a one-day workshop with experts in healthcare research. The latter two stages involved constructing a community of practice - identifying colleagues who could share perspectives on the design, conduct and reporting of case studies to explore context and complexity in health research and ensuring breadth and depth of views and types of engagement with case studies, context and complexity. Building on the Triple C meta-narrative review [29], this article focuses on findings about the notion and use of context from the interviews and one-day workshop, and broader engagement with the community of practice.

Workshop participants were recruited from a network of researchers and a Delphi panel (full details provided elsewhere [31]), including representatives from funding bodies, journals and policy organisations invited via multiple routes, including one author’s (TG) Twitter network of 100,000 individuals. Our aim was to provide maximum variation in diversity of disciplines, settings, sectors and experience. There were 58 participants based in Europe, US, Canada, Australia and New Zealand, involved in researching, evaluating, publishing, funding and developing policy and practice from case study research. The hybrid workshop comprised presentations, short provocations, and group discussions with an overall aim to help develop guidance on case study research, context and complex interventions. Small group discussions were split across four themes based on areas of debate emerging from the Triple C study at that point: ‘case study methodology’, ‘causal inference’, ‘generalisability and transferability’, and ‘operationalising context’.

In parallel to the workshop, we conducted nine interviews, including purposively sampling four authors of differing empirical case studies that we had identified in our Triple C review. Authors were invited to an online interview to understand the dilemmas and decision-making processes researchers made in designing, conducting and reporting their case studies, recognising that the content of published articles would be unlikely to represent the full story of doing case study research in practice. Interview topics were designed using our findings from the meta-narrative review [29], providing a loose structure for these discussions, including how the case and context was understood, how conceptualisations of context were operationalised into methods and analysis, and reflections on their design choices for knowledge production. The remaining five interviewees were identified through informal networks to offer insights from their different roles as journal editors, policymakers and members of funding panels engaging in research on case study, context and complexity. All interviewees were asked how to resolve some of the difficulties they had identified working with ‘context’ in their research. As is common with qualitative interview studies, rather than carrying out pilot interviews, we learnt how to adapt the design of our questions as the number of interviews progressed and our understanding of participant’s perspectives developed [32].

Discussions at the one-day workshop and all interviews were audio-recorded and transcribed verbatim. Informed by Dilley’s notion of context as an articulated set of connections and disconnections set within a social-historical context [1], we aimed to understand how participants constructed and connected context in relation to complex interventions within their discussions of case study research, as well as the wider social forces structuring these versions. Inevitably, workshop participants and interviewees did not confine their perspectives of context and interventions to case study evaluations alone, instead drawing on their experience to present arguments which cut across a range of approaches to health evaluations. Transcripts were therefore analysed to identify sequences where speakers made connections and disconnections between context and complex interventions specifically in relation to case study evaluations or health evaluations more broadly, with a focus on what was made visible in these constructions and how they oriented to wider structural forces shaping contexting practices. Ethics approval was from the Medical Sciences Inter-Divisional Research Ethics Committee (IDREC) at the University of Oxford.

## Results

Participants in our interview and workshop discussions constructed knowledge-making practices about context as shaped by epistemic and political forces, manifesting as: 1) tensions between articulating complexity underpinning context-intervention interactions and clarity of description; 2) ontological incoherence between conceptualisations of context and methods; and 3) reified versions of context being privileged when communicating with funders, journals, policymakers and publics. We present findings on each of these phenomena below, drawing on extracts from interviews and workshop discussions to exemplify how participants constructed different contexting practices and to expose structural forces shaping the production of knowledge about context and complex interventions. To help elucidate these different theoretical and methodological points we have also included illustrative exemplars from case study literature, selected to provide concrete examples of contexting practices from empirical research.

### Negotiating definitions of context and dynamic context-intervention interactions

The importance of providing definitions of what researchers meant by context in their particular studies was a central concern that all participants oriented to in interviews and workshop discussions. Some participants argued strongly that explicit definitions of context are essential for communicating how researchers framed their research and for assessing wider transferability of study findings. For one interviewee this was expressed in terms of enabling users of the research to identify whether context or the intervention required modifying to optimise its effectiveness in their own setting:

*“Then the challenge I think is actually then, you know, as a, for the reader of your work, is okay well I don’t work in Case A Case B Case C or Case D, I work, you know, somewhere else in a completely different context, so how is that learning transferable, do I have to replicate something of the context for this intervention to work most effectively or actually do I need to adjust the intervention to fit the context with which I’m, I’m in. And the whole thing is we know with complex context is that, you know, we study that complexity that I think we only ever, let’s be real we only ever scrape the surface of that complexity in terms of understanding why something works and when and how and what.”* (Funding panel member, senior researcher, journal editor)

In calling for researchers to clearly communicate elements of context and intervention, this interviewee introduces these concepts as demarcated analytical categories whilst acknowledging the complexity of why and how an intervention works in practice. Despite this acknowledgement, clarity in exposition of context and intervention as bounded objects was seen as a priority and needed to provide clear directions for change. In doing so the researcher’s talk functions to polarise categorical definitions of context and intervention from complexity rather than positioning the two concepts as potentially compatible.

Invoking the same epistemic tension between definitions of intervention-context and complexity, several participants offered a different position, which has already been identified elsewhere [17], that any attempt to separate context from intervention is fraught with difficulty:

*“I think my response might be that context and interventions are generally co-constitutive that, that, that you can’t completely, they’re interdigitated in such a way that it is very difficult to dissect them out like tumour and host they contain elements of each other”* (Senior researcher, clinical strategy advisor, clinician)

Here the interviewee invokes biomedical concepts of cancerous disease as a metaphor to construct the idea of context and intervention as intertwined, inextricably linked and therefore inherently difficult to separate. The notion that each contain elements of the other is important here, to separate is to lose something of the quality of context, as expressed by this participant in reference to conceptual frameworks of context:

*“… if you reify context as a thing then you, you start, you try and categorise, I hate those diagrams of, you know, external context, internal context, this context, that context, no, no, no, no it’s just all stuff of what, you know, the, there’s a, I, I think [um] and I think, I think one of the, one of the issues with, with trying to define it is that you, whatever, however you do it you might, you start losing things”* (Senior researcher, clinician)

**Fig. 1:**
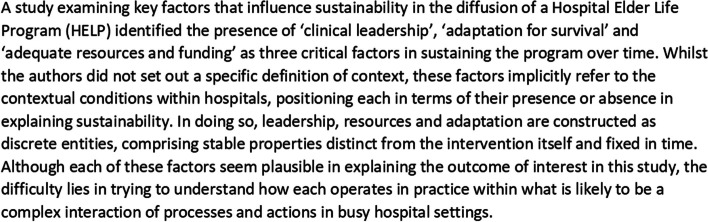
Categorising context as distinct from the intervention

Instead of definitions of context functioning to enhance our understanding of intervention-context interactions, the opposite occurs, with the researcher’s purposive selection of the relevant context inevitably excluding something else, in effect rendering it invisible. Figure 1 provides an example of this, drawn from a case study of an intervention for elderly patients in a hospital setting [33].

One interviewee specifically focused on leadership as an example of abstracting context in a way which is far removed from the social phenomena being investigated:

*“… that just leaves me as a researcher really wanting to use and build on the work that they’ve done with a lot of work and unpacking what does this even mean and depending on the methodology I’m not at all sure that I can understand what they mean by leadership, I mean because these labels or these categories that are sometimes very far removed from what I would expect was the very messy complex practice that they were studying.”* (Senior researcher)

In this version, abstractions of context and intervention are distanced from the *“messy complex practice”* of implementation. To reduce this distance requires analytical reframing from generic categorical definitions of context as a list of ‘things’, to specific ontologies which meaningfully connect with implementation practices, for example as processes, social actions or interactions. In doing so, the researcher is required to specify what they have counted as empirical evidence of concepts such as leadership in their ‘contexting practices’, as well as the methods and analytical approach they have employed to foreground particular versions of context whilst backgrounding others.

### Ontological (in)coherence in research methods for investigating context

A second way in which experts oriented to problems of context and visibility was how researchers engage with key principles underpinning qualitative research, specifically the connection between ontology, methodology and method:

*“I’m always thinking about the methodology and what, how, what am I doing to be able to, what, what is what I’m doing allowing to express how I’m viewing this data and what my view of the world is and why I think that you need to look at it in this way because any piece of data in my view can be analysed in lots of different ways you just need to decide what that question is and what that question requires. So does that question require you to use a very in-depth method like conversation analysis or actually is the question you answered doesn’t actually require that level of interactional data, what it actually requires is understanding what people think and feel or, you know, or what they’re saying, you know what, what are people’s accounts, what is it we’re trying to understand.”* (Senior researcher, journal editor)

Picking up on this call for greater methodological specification, another participant argued that understanding complex and dynamic interactions requires an ontology of relations:

*“One of the hardest things is actually to understand and to write about, theorise the relationality which is constitutive of the case, of the context, of the researcher, of the interactions between these different things, but it’s in the relationality that the dynamics of change actually take place.”* (Senior researcher)

**Fig. 2:**
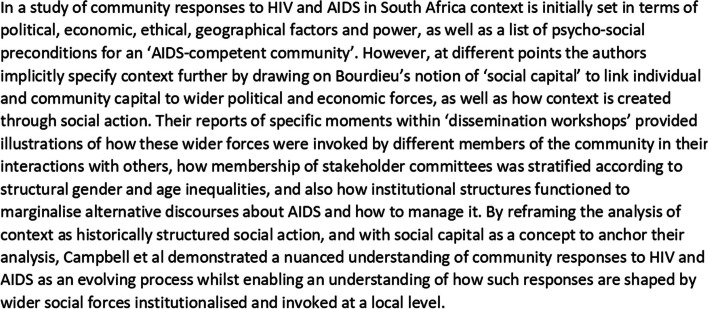
Mobilising context to enhance visibility of spatial and temporal forces

In contrast to the tensions we presented earlier between context definition and complexity, this researcher constructs relationality as the mechanism for revealing complex and dynamic interactions between context and other phenomena. This has important implications, requiring a data collection method and analytical approach that facilitates insight into such relationality. Figure 2 provides an example, from a study by Campbell et al [34] of community responses to HIV in South Africa, of what is afforded - made visible - by such an approach, seen here in terms temporal and spatial contexts.

Ontological coherence within health evaluations necessitates an explicit link between how context has been conceptualised and how it manifests empirically. By conceptualising context as emergent through socially structured action, Campbell et al constructed an ontology which required data and a level of analysis which exposed this relationship. The analysis of actions within dissemination workshops provided ontological coherence between theory and method, viewed not simply as actions of individuals, but as moments invoking wider social forces. In doing so, they generated an understanding of context across time and space that might otherwise been invisible.

### Privileging versions of context in knowledge production and dissemination

In the previous sections we have discussed different threads in the production of knowledge about context according to how researchers construct theoretical relations between context and interventions, and how conceptualisations of context are translated and ‘made’ through choice of methodology, methods and analysis. In this section we focus on the different ways researchers negotiate these difficulties, which arguably reflects how research about complex healthcare interventions has institutionally privileged certain types of research design and knowledge about context over others. In the Triple C study, we have found that this contrasts with a careful consideration of the constructive process in capturing context-intervention relations as emergent, complex and dynamic. In one workshop discussion several participants reflected on the consequences of this privileging work:

*“I think it’s important and then as others have said I think it’s quite helpful to think about, because we’re talking about all of these things, context of the intervention, context of the phenomenon which can change the context of the research, so those are sort of three ways and, you know, you spoke to that. And then I think as you were just saying [name] and then I think [name] as well, what knowledge is getting privileged, right, and so we could still apply that to context, like what are the unintended consequences therefore and shining a light on what knowledge about context is being privileged and therefore how a case gets framed”* (Senior researcher)

The notion that particular versions of context come to be privileged over others shifts the intervention-context dynamic from an empirical and analytical concern to one that is epistemic and political. One way this was evident in our review was how authors frequently placed limitations on the explanatory power of their case study findings. Positioned as offering transferability rather than stronger claims to conceptual or theoretical generalisability, these mitigating statements potentially functioned as a response to arguments from journal editors and reviewers that small-scale, N=1 case studies are too context-specific to facilitate wider generalisability.

In contrast, experts in our interviews and workshop discussions oriented to the pressure on researchers to provide clear messages to funders, policymakers and publics. As we discussed earlier in this article, this pressure revealed a specific tension between communicating the complexity of implementation against a need to provide clear messages to users of the research. One interviewee reflected on this from their experience as a director of clinical strategy:

*“I speak as somebody who would have been the recipient of those reports previously, I would, I do not, in those days I did not want to hear all the, you know, complexities and nuances and blah, blah, blah, blah, blah I just wanna know what did you do, what are your recommendations give me the recommendations and actually I wouldn’t have read the whole thing I would have read the executive summary, the executive summary, what are the recommendations, handed it to somebody and said go and do that. I’m very changed now, I’m changed”* (Senior researcher, director of clinical strategy, clinician)

Here, this interviewee constructs a before and after story, where they had changed their view from wanting simple clear recommendations to one on the importance of communicating complexity for healthcare improvement “*without getting lost in the language of complexity theory.”* A response offered to this challenge by several participants was the explanatory power of illustrative examples or stories. Below, another interviewee positions stories as the vehicle for bridging a divide between complexity inherent within naturalistic case studies [30] and the push to produce concrete recommendations:

*“But I do think that there’s a real gap between* [naturalistic case studies] *and the hardcore evaluation type work that I try to bridge. So it would be incredibly helpful to try and work out ways of making that transition, guiding that transition or, you know, because it’s almost like what does the MRC need, it needs guidance to help policymakers, and policymakers will want particular types of data, we know they don’t like numbers so then you talk about context and they do actually love case studies, they love stories, they love narratives”* (Senior researcher)

Another emphasised the constructive process in using stories to communicate complexity to government ministers, involving decisions about which version of the story gets told:

*“you have to make prioritisation decisions about what’s important and what isn’t and it’s not, and, and in grappling with the complexity it’s not just the dramatic that’s important, the ordinary might be important to carry on with the, play with the metaphor, the sort of kitchen sink drama, you know, is an important part of it [laughs] of the narrative.”* (Policymaker, researcher)

The construction of context for a journal audience and the construction of context for policymakers position the research as specific, detailed and involving situated accounts. Yet the contrast is stark in terms of their explanatory power. In the former, context as small-scale functions to constrain wider applicability beyond the confines of the research that has been completed; in the latter, a story used to articulate complexity of the context is positioned as a rhetorical device for wide-scale change at a policy level.

**Fig. 3:**
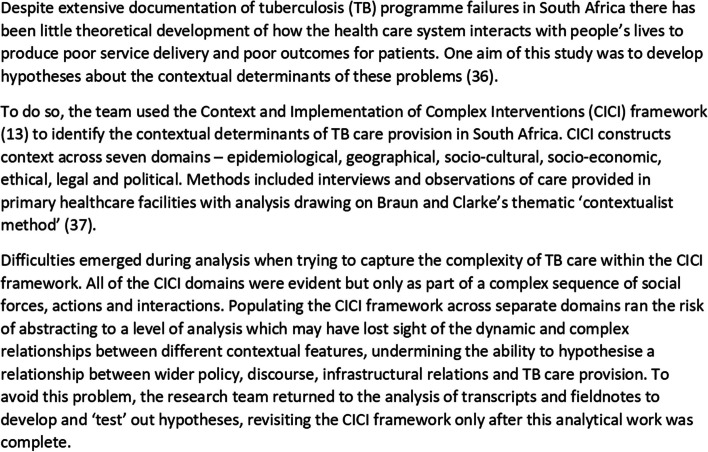
Using contextual determinant frameworks to structure investigations [13, 36, 37]

Figure 3 provides a reflective example of these epistemic and political forces, taken from a study involving one our Triple C team (JM). Here the construction of context was shaped by funder’s expectations to produce evidence on the utility and performativity of Implementation Science frameworks for ‘taming’ context in complex health interventions. The study comprised eight separate work packages under an over-arching objective to strengthen health systems in sub-Saharan Africa [35]. Each work package focused on a different population and topic but with a specific remit to identify the contextual determinants of problems in healthcare delivery; and to develop, implement and evaluate an intervention to address those problems. Importantly for this article, a cross-objective required by the funder was to develop recommendations on the use of Implementation Science frameworks for health systems strengthening in low and middle-income countries. As Fig. 3 illustrates, the use of a determinant framework in one of the work packages led to an analytical process involving difficult negotiations of visibility in context-intervention interactions.

## Discussion

The different ways researchers engage with contexting within projects, or as journal editors, funders and policymakers, exposes a mutually constructive process between researcher, institutional forces and audience that typically privileges certain versions of context over others. This can be seen in terms of how context is formulated to guide investigations and subsequently operationalised into research design, through to how context is used to legitimise findings and influence policy and practice. What follows from this is the notion of context as-a-thing ‘out there’, as research object to be discovered and as distinct from a health intervention, is problematic.

Across our interview and workshop discussions context was often conceived in complex and dynamic interactions with interventions. Yet while this notion is sometimes formulated as a theoretical construct in empirical literature, it is infrequently deployed in research designs, failing to thread this ontology of context into research methods, analysis and findings. Evaluations appear to uncritically defer to interview methods, deductive frameworks and thematic analytical approaches, arguably limiting the potential for capturing complex interactions over time. Fabian recognised this limitation in anthropological research as long ago as 1983, arguing that fieldwork constructs a timeless “ethnographic present” that “freezes a society at the time of observation” [38]. Yet such critical insights on the relationship between method and knowledge production appear to be lost in health evaluations, and unsurprisingly the findings produced through this process struggle to mobilise the complexity originally conceived, producing lists of contextual conditions or triggers for optimising implementation, distinct from the intervention itself. Within academia, it is this version of context that appears to have the greatest traction for obtaining funding and publication, supposedly offering clear and concrete outputs for others to comprehend and use for their own purposes. Paradoxically, for policymakers it may be the highly contextualised narrative that has the greatest utility and potential for change. Whilst we are offering crude distinctions here, the production of knowledge about context can be seen as a social and epistemic practice, shaped and reshaped along a trajectory, negotiating tensions on that journey between concerns of specificity and complexity with concerns of structure, clarity and potency.

The reasons why complexity and dynamism of context-intervention interactions are often acknowledged, but rarely materialised, in empirical studies are difficult to disentangle. It requires widening the focus beyond the design choices made by individuals within individual studies, to an understanding of how different disciplines have shaped health evaluations over this period. Understanding the emergence of complex systems thinking may be important here which, in itself, has functioned as an innovation that has disrupted long-established epistemic traditions perpetuating positivist assumptions of objectivity, linear causality and predictability. Within this view, the talk of participants in our study exposed this disruption, and echoing Dilley’s ‘dilemma of context’ [1], these interactional moments illustrate colleagues grappling with a nuanced appreciation of context within a dominant discourse which privileges a reified version of context. They also provide windows into methodological developments in health research that enable researchers to attempt to navigate the space between ideologies of context as reified category and context as emergent, fluid and complex. Arguably, the emergence of Implementation Science in the last 20 years represents one such development, situating an understanding of context as a central ‘real-world’ concern whilst promoting the use of structured frameworks that run the risk of glossing out the particularities of implementation. Another development perhaps lies in the emergence of ‘programme theories’, which bound context within the immediate conditions interacting with the intervention, or ‘middle-range theories’ that attempt to straddle broader social theory with programme theories.

One consequence of these developments has been a proliferation of health evaluation publications that infrequently draw on a rich and diverse body of critical thinking about qualitative research methodology. As individual researchers working within these wider epistemic practices and as part of large research teams, we acknowledge that we have also been part of this proliferation, reproducing many of the problems identified in this paper. Moreover, we are likely to continue to reproduce these problems given that we continue to be bound by the same institutional and structural expectations. Critical thinking in qualitative research requires the researcher to reflect and investigate their assumptions and position in relation to the object of investigation, provides methodologies which enable investigation of these assumptions, and the methods, data and analysis that operationalise these methodologies [39, 40]. As we summarised in the background, a number of articles have already argued how it might be more fruitful to shift the focus away from context as a distinct category to an analytical focus on the social practices, actions, interactions, processes, forces or entanglements which enact an intervention. These different framings of context will have distinct nuances in their ontological underpinnings but they each ask us to think about how we might observe and analyse forms of context as something that happens and as something that researchers do (verb), rather than context as something that is (noun).

Reconfiguring context in this way – i.e., as something that happens - opens up opportunities to draw on a wealth of methodologies, methods and analytical techniques across disciplines, offering different ways of doing visibility within health evaluations. Some of these approaches are well-known and widely used in health research, including ethnographic observations of socially structured actions involved in implementation [41], or conversation analysis [42], which reveal participants’ orientation and negotiation of relevant meaning through ongoing interaction. However, we contend that a rich body of theories, methodologies, methods and analytical techniques have been available across disciplines for some time and that can provide powerful ways to mobilise context in health evaluations. To date, these appear to have been largely ignored.

Here we suggest ways to engage with these theoretical and methodological resources to enable a more dynamic conception and operationalisation of context, recognising that these suggestions are by no means comprehensive. An obvious place to start is to draw on the range of approaches that fall under the umbrella of complexity sciences and systems thinking. In 2018, Brian Castellani [43] produced a timeline and map of the complexity sciences from the 1940s to the present day, linking perspectives on complexity across diverse disciplines including biology, cognitive science, mathematics, data science, geography and organisational studies to name but a few. Whilst approaches to complexity are diverse and also include reductionist models, underpinning assumptions of emergence and unpredictability provide a foundation for theorising and operationalising context and intervention as inextricably linked and involving complex interactions over time.

Another rich resource that has been under-utilised in health research can be found in discourse analysis [44], sociolinguistics [45] and linguistic ethnography [46], which provide conceptual and analytical tools for understanding context invoked through text, talk and signs, arguably the central mechanisms by which implementation is enacted. Intervention-context interactions may be made visible by mapping which discourses are in place within and across the different events involved in implementation, how different discourses interact through text and talk, their function and the consequences for implementation. Language and history as units of analysis are critical here, how each event emanates from previous events but also anticipates future events. Through analysis of discourse, we can retrospectively or prospectively evaluate intervention delivery by analysing the intersection of discourse and action at specific events and tracking the sequence of events over time (e.g., [47, 48]). Other approaches may place more emphasis on the use of interventions as ‘sociomaterial practices’ through which interventions are enacted [49], for example by analysing the socio-technical networks which connect human and non-human entities which give the intervention ‘its local colour’ of intervention-context interactions [50, 51].

At stake here is whether these different approaches to context ultimately lead to meaningful change for the diversity of people the health interventions are intended to benefit. Understanding what users of interventions need to know about context-intervention interactions to enable decisions about implementation across a wider range of settings requires systematic engagement and open dialogue which links health practice, health policymaking and academic analysis. These conversations are inevitably shaped by political concerns and epistemic conventions for driving impact from research evidence. It is therefore crucial that researchers collaborate with these users to work out who are most likely to benefit from a more complex understanding of context-intervention interactions, how this might happen and how this version of context represents an advantage over simply producing a list of conditions for users to replicate in different settings.

At this point it is helpful to return to the opening quote and what Dilley referred to as “the politics of context definition”, articulated through socially and historically situated connections and disconnections for particular purposes. In keeping with this understanding, we are conscious that our ‘findings’ are accounts that we have co-constructed through a dialogue with empirical papers and colleagues involved in our discussions. There are three issues that are important to acknowledge here. Firstly, the study team, workshop participants and interviewees comprised many colleagues with a strong track record problematising complexity and context. Given that, it is perhaps no surprise that participants oriented to critical perspectives that challenge context as a reified category distinct from the intervention, revealing their ontological, social, and political positions that conflict with a wider biomedical discourse and practices within which they work. Secondly, whilst we sought the inclusion of participants across the globe, there was a notable absence of participants from the Global South. It is therefore possible that we did not obtain insight into other important narratives which connected context with case studies and complexity. Finally, our discussions took place within a study with a specific focus on case study methods. Whilst participants unavoidably drew on their wider experience of health research when expressing views on context, these conversations might have differed if *we* had oriented to the role of context in health research more widely. Nevertheless, the dissonance we found between how context was talked about, and how context was written about, speaks to narratives of context that have been discussed elsewhere [28], raising fundamental questions about the processes of knowledge construction to tackle complex social problems within public health and health systems research more widely.

We do not intend to prescribe how health researchers and users of research should engage with context in health research – as we have shown here each individual acts as an author within a wider system of knowledge production, engaging with different reconstructive processes of context for different purposes. However, we do assert that if the intention is to understand complex and dynamic intervention-context interactions, and if we are to acknowledge the stories that get told about context in achieving that end, then greater critical reflection and transparency on how context is being deployed along the research trajectory is required, from how context is conceived through to dissemination. We call for health services researchers to connect with how scholars approach complexity and context across disciplines. To do so provides opportunities for creatively changing the field in which health evaluations are conducted, enabling a critical standpoint to long-established traditions and opening up possibilities for innovating the design of evaluations of complex health interventions.

## Conclusions

To date the importance of understanding complex and dynamic intervention-context interactions in health evaluations has been widely acknowledged. However, our findings suggest that there has been limited success in translating this conceptualisation into evaluations of complex health interventions. Facilitating such understanding may be strengthened by increasing the diversity of disciplines for investigating context in health research. Ontology and epistemology lie at the heart of this work, including a consideration of how context-intervention interactions might be mobilised as historically situated social events or practices. It is not sufficient for researchers to change how they engage with context on an individual level. Context as distinct from interventions has been the privileged version dominating how health research has been funded, conducted and disseminated, shaped by long-established academic conventions in this field, which overwhelmingly operationalise context in this way. We call on researchers, policymakers, funders and editors to recognise that a change in the field of health research is urgently required, involving wide-scale critical engagement of the historical forces that have shaped the production of knowledge about context and interventions, and importantly how this has been – still is - consequential for our ability to effect meaningful change.

## Data Availability

The datasets generated and/or analysed during the current study are not publicly available due to data transcripts including participant information not suitable for sharing but are available from the corresponding author on reasonable request.

## Abbreviations

CICI: Context and Implementation of Complex Interventions framework
